# Skin, mucosa and nail findings in hospitalized pediatric patients with Coronavirus disease-2019 (COVID-19)^☆^

**DOI:** 10.1016/j.abd.2022.03.006

**Published:** 2022-11-23

**Authors:** Tunç Özen, Filiz Cebeci Kahraman, Sevliya Öcal, Hüsnü Fahri Ovalı

**Affiliations:** a Department of Dermatology, İstanbul Medeniyet University, Göztepe Prof. Dr. Süleyman Yalçın City Hospital, İstanbul, Turkey; b Department of Pediatric Infectious Diseases, İstanbul Medeniyet University, Göztepe Prof. Dr. Süleyman Yalçın City Hospital, İstanbul, Turkey; c Department of Pediatric Diseases, İstanbul Medeniyet University, Göztepe Prof. Dr. Süleyman Yalçın City Hospital, İstanbul, Turkey

**Keywords:** Child, COVID-19, Mucous membrane, Skin manifestations

## Abstract

**Background:**

Cutaneous manifestations of Coronavirus Disease-2019 (COVID-19) disease have not yet been fully described in hospitalized pediatric patients.

**Objectives:**

This prospective study aims to demonstrate the skin, mucosal, and nail findings of hospitalized children with COVID-19.

**Methods:**

The authors included hospitalized pediatric patients. Two dermatologists examined skin, hair, nails, and mucosa. Patients with drug eruptions were excluded with an anamnesis, clinical and laboratory test results.

**Results:**

Out of 46 enlisted patients, 19 (41,3%) patients displayed skin, mucosal or nail findings. Skin findings were seen on 14 (30.4%) patients. Ten (22%) patients presented skin findings matching described patterns. Half of the patients with patterned rashes had confluent erythematous/maculopapular/morbilliform rashes. Eleven out of 46 (23.9%) patients developed periorbital erythema and edema. Ten (22%) patients had at least one oral mucosal finding. One telogen effluvium, one blue nail, and one flag sign on nails were noticed. Nine (19.5%) patients out of 46 had developed MIS-C. MIS-C patients had mucocutaneous manifestations except one (88.8%).

**Study limitations:**

The authors have detected a higher rate of mucocutaneous manifestations compared to out-patients with mild COVID-19 because the study is based on hospitalized patients only.

**Conclusions:**

Pediatric COVID-19 patients are more susceptible to developing mucocutaneous manifestations compared to adults. The authors propose COVID-19 should be acknowledged as one of the viral exanthem rashes of childhood. The authors noticed that the most common findings were periorbital erythema and edema. The confluent erythematous/maculopapular/morbilliform rashes appear to be the most common patterns associated with severe COVID-19.

## Introduction

A novel coronavirus denominated SARS-CoV-2 precipitated a pandemic on 31 December 2019, which has led to a 5.4 million death toll according to the WHO (World Health Organization) statistics on 02 Jan 2022. At first, the disease was identified by respiratory findings, but subsequent reports declared that Coronavirus Disease-2019 (COVID-19) could involve any system such as that cardiovascular, integumentary, immune, and neurological systems.^1^ Early reports proclaimed that pediatric patients have a milder disease course compared to adults.^2^ The definition of a novel syndrome called MIS-C (Multisystem Inflammatory Syndrome in Children) challenged this assumption. In literature, many reviews and studies have defined the skin findings of COVID-19 by patient consultations which were seen via teledermatology and social media platforms. Nevertheless, the authors did not encounter a prospective study in which the hair, nail, skin, and mucosa of pediatric COVID-19 inpatients are thoroughly documented by a dermatologist. This study aims to shed light on the skin, mucosal, and nail findings of hospitalized children with COVID-19.

## Methods

### Study design and participants

The authors performed a prospective study from January 2021 to April 2021. Ethical approval was obtained from the local ethics committee (date: 13.01.2021, number: 2021.0022). Informed consent was obtained from all participants. The study group consisted of patients aged under 18 years, who were staying in pediatric infectious service. Patients with suspected adverse drug eruptions were excluded using the following three criteria: (i) Typical clinical presentation, (ii) Blood and tissue eosinophilia and/or clinical-pathological correlation, (iii) No history of suspicious drug use. Two dermatologists examined the skin, oral mucosa, and nails of 46 patients with MIS-C or COVID-19 twice weekly. During each visit, a form, which outlined details about the patient’s mucocutaneous rash, was filled and mucocutaneous findings were photographed. A COVID-19 PCR test, chest X-Ray imaging, or a COVID-19 antibody test was performed by the pediatric infections department for the diagnosis of COVID-19. The authors performed two punch biopsies in course of the present research. The reason being is that those two patients with the diagnosis of MIS-C had manifested severe cutaneous rash. Informed consent was obtained from the guardian of the children. Using the Center for Disease Control and Prevention case definition of MIS-C, patients were included if they met the following criteria: (1) Aged 21 years or younger presenting with fever, laboratory evidence of inflammation, and severe illness requiring admission; (2) Had involvement of at least two organ systems; and (3) Had no alternative plausible diagnosis.^3^ The skin findings were categorized into clinical patterns, which were previously defined by Giovanni Genovese et al.^4^ The clinical patterns encompass (i) Urticarial rash, (ii) Confluent erythematous/maculopapular/morbilliform rash, (iii) Papulovesicular exanthem, (iv) Chilblain-like acral pattern, (v) Livedo reticularis/racemosa-like pattern (vi) Purpuric “vasculitic” pattern.^4^ The authors divided confluent erythematous, maculopapular, and morbilliform rash into separate groups.

### Outcomes

The outcome of the present study was to identify COVID-19 induced skin, mucosa, nail, and hair findings and determine their rates.

### Statistical analysis

Descriptive statistics were presented as numbers and percentages for categorical variables and mean for numerical variables.

## Results

**Figure 1:**
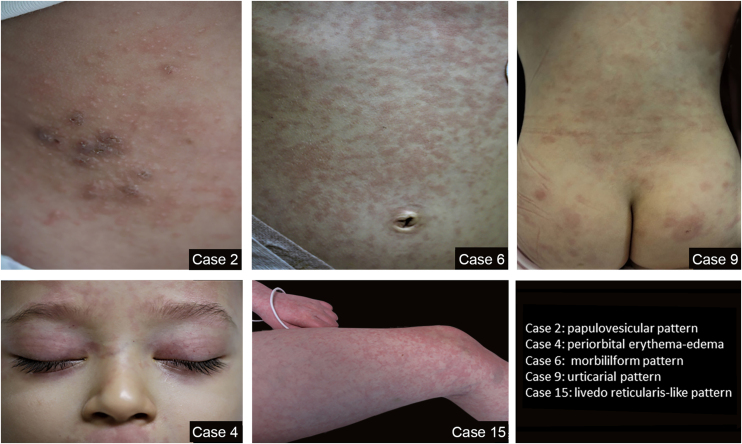
Some of the patterns of rashes in the study group.

The study group included 46 patients with 21 (45.6%) females and 25 (54.6%) males (a mean age of 8.5 years and an age range of 2 months – 17 years). Out of 46 enlisted, 19 (41.3%), patients displayed skin, mucosal or nail findings. Among 19 patients with mucocutaneous findings, 13 were female and six were male (the mean age was 5.2 and the age range was 3 months – 17 years). The mean ages of the female and male patients with mucocutaneous findings were 5, 5.6, respectively. Skin findings were seen on 14 (30.4%) patients. Ten (22%) patients presented skin findings matching the previously discussed patterns with three maculopapular, one morbilliform rash, one confluent erythematous, two urticarial rash, two papulovesicular exanthem, one livedo reticularis/racemosa-like pattern. Half of the patients with pattern rashes belonged to the confluent erythematous/maculopapular/morbilliform rash group, which appears to be the most common pattern. Other skin findings are comprised of eleven periorbital erythema and edema, one acral erythema, and one periungual desquamation. 23.9% of the study group developed periorbital erythema and edema, which was the most striking skin finding. In eight patients, pattern rashes were accompanied by periorbital erythema and edema and out of those eight patients in one patient, there is concomitant acral erythema. Only three patients exhibited periorbital erythema and edema without any other skin finding accompanying. Characteristics of rash patterns and other skin findings of the study group are present in Table 1. Some of the patterns of rashes in the study group are seen in Fig. 1 A,B,C,D,E.

**Table 1:** Rash patterns and other skin findings in the study group.

Clinical characteristics n (%)	Male (n = 25)	Female (n = 21)	Study group (n = 46)
**Rash patterns**	6 (13%)	4 (19%)	10 (21.7%)
Maculopapular	2 (4.3%)	1 (2.1%)	3 (6.5%)
Morbiliform	1 (2.1%)	‒	1 (2.1%)
Confluent erythema	‒	1 (2,1%)	1 (2.1%)
Livedo reticularis like	1 (2.1%)	‒	1 (2.1%)
Urticarial	‒	2 (4.3%)	2 (4.3%)
Papulovesicular	2 (4.3%)	‒	2 (4.3%)
Chilblain-like	‒	‒	‒
Purpuric “vasculitic”	‒	‒	‒
**Other skin findings**			
Periorbital erythema and edema	6 (13%)	5 (10.8%)	11 (23.9%)
Concurrent with a rash	5 (10.8%)	3 (6.5%)	8 (17.3%)
Not concurrent with a rash	1 (2.1%)	2 (4.3%)	3 (6.5%)
Acral erythema^a^	‒	1 (2.1%)	1 (2.1%)
Periungual desquamation	‒	1 (2.1%)	1 (2.1%)

a Acral erythema is accompanying one maculopapular rash.

**Figure 2:**
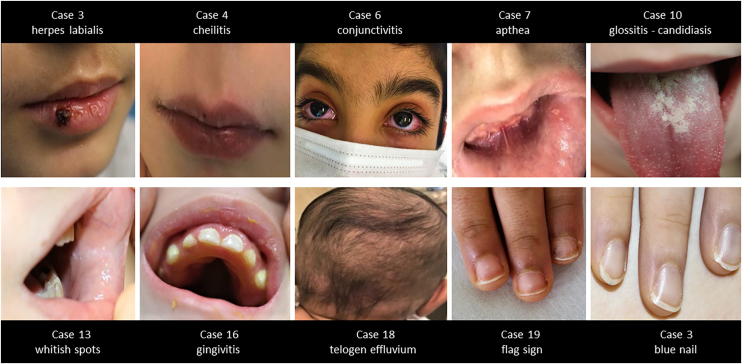
Some of the mucosa, nail and hair findings of the study group.

Mucosa examination revealed six cheilitis, five conjunctivitides, two glossitis (strawberry tongue), two oral aphthae, two whitish plaques on the buccal mucosa, two oral mucosal candidiasis, one herpes labialis, one gingivitis. Ten (22%) patients had at least one oral mucosal finding. During hair and nail examination one telogen effluvium, one blue nail, and one flag sign on nails were noticed. Mucosa, nail, and hair findings in the study group are seen in Table 2. Some of the mucosa, nail, and hair findings of the study group are seen in Fig. 2 A,B,C,D,E,F,G,H,I,J.

**Table 2:** Mucosa, nail and hair findings in the study group.

Clinical characteristics n (%)	Male (n = 25)	Female (n = 21)	Study goup (n = 46)
**Mucosa findings**	3 (6.5%)	7 (15.2%)	10 (21.7%)
Conjunctivitis	3 (4.3%)	2 (2.1%)	5 (10.8%)
Cheilitis	3 (6.5%)	3 (6.5%)	6 (13%)
Glossitis	1 (2.1%)	1 (2.1%)	2 (4.3%)
Oral aphthae	‒	2 (4.3%)	2 (4.3%)
Whitish spots on the buccal mucosa	‒	2 (4.3%)	2 (4.3%)
Oral mucosal candidiasis	2 (4.3%)	‒	2 (4.3%)
Gingivitis	‒	1 (2.1%)	1 (2.1%)
Herpes labialis	1 (2.1%)	‒	1 (2.1%)
**Nail findings**	1 (2.1%)	1 (2.1%)	2 (4.3%)
Blue nail	1 (2.1%)	‒	1 (2.1%)
Flag sign	‒	1 (2.1%)	1 (2.1%)
**Saç bulguları**	1 (2.1%)	‒	1 (2.1%)
Telogen effluvium	1 (2.1%)	‒	1 (2.1%)

Nine (19.5%) patients out of 46 had developed MIS-C. Each of them presented pulmonary involvement with systemic end-organ involvement such as cardiovascular and renal involvement. MIS-C patients had mucocutaneous manifestation except one (88.8%). Patients with MIS-C exhibited substantially more skin and mucosal findings than patients without it. The most striking manifestations of nine MIS-C patients were seven periorbital erythema and edema, five conjunctivitis, and/or five cheilitis. The most frequent patterns the authors encountered in this group were two maculopapular rashes, one morbilliform rash, and one confluent erythema. Age, sex, initial systemic manifestations, patterns of the lesions, the presence of MIS-C, result of the PCR test, and treatments of cases with COVID-19 with skin, hair, and nail findings are seen in Table 3. Three patients developed skin manifestations simultaneously with COVID-19 symptoms such as fever, asthenia, myalgia, arthralgia and respiratory symptoms. In 14 patients the onset of mucocutaneous findings was followed by the onset of COVID-19 symptoms. Two patients showed skin manifestations prior to COVID-19 symptoms.

**Table 3:** The characteristics of coronavirus disease-2019 cases with a skin, nail, hair, and mucosa finding.

Case number	Age/Sex	Initial systemic manifestations	Patterns of the lesions skin	Mucosal lesions	MISC	PCR	Treatment
1	F/3¥	Fever, myalgia, arthralgia, respiratory symptoms, asthenia	Papulovesicular pattern rash	None	No	+	Mupirocin 2% ointment, Symptomatic treatment
2	F/8	Asthenia	Papulovesicular pattern rash	None	No	N/A	Hamamelis virginiana extract ointment
3^a^	M/8	Fever, myalgia, arthralgia, respiratory symptoms, asthenia	Maculopapular pattern rash, periorbital erythema and edema	Conjunctivitis, glossitis (strawberry tongue), herpes labialis, cheilitis, candidiasis on the tongue	MISC	‒	Methlyprednisolon (30 mg/kg/day), IVIG (1 g/kg/day), aspirin
4	M/2	Fever, myalgia, arthralgia, respiratory symptoms, asthenia	Maculopapular pattern rash, acral erythema, periorbital erythema and edema	Conjunctivitis, cheilitis	MISC	+	Methlyprednisolon (10 mg/kg/day), IVIG (1 g/kg/day, 3 days)
5	M/1	Fever, myalgia, arthralgia respiratory symptoms, asthenia, dysgeusia	Maculopapular pattern rash, periorbital erythema and edema	No	No	+	Methlyprednisolon (10 mg/kg/day), IVIG (1 g/kg/day, 3 days)
6	F/4	Fever, myalgia, arthralgia respiratory symptoms asthenia, dysgeusia	Morbiliform pattern rash, periorbital erythema and edema	Conjunctivitis, cheilitis	MISC	+	Methlyprednisolon (30 mg/kg/day), IVIG (1 g/kg/day, 5-days)
7	F/4	Fever, myalgia, arthralgia, Respiratory symptoms asthenia	Confluent erythema, Periorbital erythema and edema	Conjunctivitis, Oral aphthea	MISC	+	Methlyprednisolon (30 mg/kg/day), IVIG (1 g/kg/day, 3 days)
8	M/8	Fever, arthralgia, myalgia, respiratory symptoms asthenia	Urticarial pattern rash, periorbital erythema and edema	None	No	+	Methlyprednisolon (30 mg/kg/day), IVIG (1 g/kg/day, 5 days)
9	F/3	Fever, arthralgia, myalgia, respiratory symptoms, asthenia	Urticarial pattern rash, periorbital erythema and edema	Cheilitis	MISC	N/A	Methlyprednisolon (30 mg/kg/day)
10	M/5	Fever, arthralgia, myalgia, respiratory symptoms, asthenia	Periorbital erythema and edema	Conjunctivitis, cheilitis, glossitis (strawberry tongue), candidiasis on the tongue	MISC	+	Methlyprednisolon (30 mg/kg/day)
11	F/8	Respiratory symptoms	Periungual desquamation	None	No	+	Topical vaseline ointment
12	F/3	Fever, respiratory symptoms	Periorbital erythema and edema	None	No	+	Symptomatic treatment
13	F/6	Fever, arthralgia, myalgia, Respiratory symptoms, dysgeusia	Periorbital erythema and edema	Whitish spots on buccal mucosa	MISC	+	Methlyprednisolon (30 mg/kg/day)
14	F/17	Respiratory symptoms	None	Whitish spots on buccal mucosa	No	+	Topical 0.2% chlorhexidine digluconate
15	M/10	Fever, arthralgia, myalgia, respiratory symptoms	Livedo reticularis-like pattern, periorbital erythema and edema	None	MISC	+	Methlyprednisolon (30 mg/kg/day), IVIG (1 g/kg/day, 3 days)
16	F/3	Fever, arthralgia, myalgia, respiratory symptoms	None	Gingivitis, cheilitis	No	+	Topical Hamamelis Virginiana extract ointment
17	F/4	Asthenia	None	Oral afthae	No	N/A	Oral nystatin mouthwash
18^b^	F/4¥	Fever, respiratory symptoms	None	None	No	+	Symptomatic treatment
19^c^	F/4	Fever, respiratory symptoms	None	No	No	+	Symptomatic treatment

F, Female; M, Male; MISC, Multisystem Inflammatory Syndrome in Children; PCR, Polymerase Chain Reaction; IVIG, Intravenous Immunoglobulin; ¥, Months.

a Besides skin and mucosal findings patient possesses a nail finding (blue nail).

b Only exhibits a hair finding (diffuse alopecia).

c Only exhibits a nail finding (flag sign).

Two of the MIS-C patients underwent a skin biopsy. The first skin biopsy showed undulation of the epidermis, neutrophils and scarce eosinophils. The second one revealed prominent vascular endothelial proliferation of dermis capillaries, few lymphocytes, scarce plasmocytes, and vasculopathy with sporadic erythrocyte extravasation.

## Discussion

The skin manifestations of adult COVID-19 patients have been clearly defined. Early data of the pandemic revealed that 0.2%^5^‒20.4%^6^ of adult COVID-19 patients developed skin manifestations. The following study illustrated that 24% of hospitalized adult patients showed skin manifestations.^7^ Current data investigating the relationship between COVID-19 and skin manifestations revealed that drug eruptions were inaccurately diagnosed as COVID-19 skin findings.^8^ Studies investigating the association between COVID-19 and skin manifestations are scarce in the pediatric population. The present study demonstrated that a substantially high percentage of pediatric COVID-19 patients showed mucocutaneous manifestations (41.3%). To the best of our knowledge, this is the first prospective study in which the hair, nail, skin, and mucosa of pediatric COVID-19 inpatients are thoroughly documented. The authors did not encounter any research that analyses patterns of rashes to provide statistical details. Therefore, the authors shaped the discussion by comparing the present findings with the skin findings of adult COVID-19 patients.

The authors noticed that the most common finding was 23.9% periorbital erythema and edema, which has so far remained under-recognized among the skin findings of pediatric COVID-19 patients. In the present study seven out of 11 (63.6%), patients showed periorbital erythema and edema, which was evaluated as periorbital erythema and edema is associated with severe COVID-19. In the study of Young et al. 20% of the MIS-C patients exhibited periorbital erythema and edema and they similarly indicated a correlation between severe COVID-19 and periorbital erythema and edema. The significance of periorbital erythema and edema is also mentioned by Terzi et al.^9^

In the present research, 10.7% of the patient population exhibited a rash categorized in the combined group of confluent erythematous/maculopapular/morbilliform rash and the combined group was the most frequent pattern among patterned rashes. If the authors examine the patterns in adult patients, studies illustrated that the combined group of confluent erythematous/maculopapular/morbilliform rash comprised the most significant group amongst the patterns with 23%‒70%.^7^, ^8^, ^9^, ^10^ Another study revealed maculopapular exanthema was identified in 47% of the adult patients 78% of whom had a history of one or more drug usage.^6^ This circumstance might be clarified by the fact that adverse drug reactions can be misdiagnosed as a maculopapular exanthema of COVID-19. A recent study revealed that confluent erythematous/maculopapular/morbilliform rash was associated with more severe COVID-19.^11^ In present case, these rashes are frequently concurrent with systemic end-organ damage and MIS-C. The pathogenesis of COVID-19 induced skin manifestations consists of the immune response against viral nucleotides, thrombotic diathesis, and drug reactions. Viral exanthema-like clinical appearance is presumed to be triggered by an immune response to viral nucleotides.^13^

The urticarial eruption was reported 10%‒20%^10^, ^12^, ^14^ of adult COVID-19 patients. In the present study, 4.3% of the patients showed urticarial eruption. Viral infections might trigger urticaria by mast cell degranulation via complement activation or vasculitis, which is precipitated by the COVID-19 virus binding to Angiotensin-Converting Enzyme (ACE) 2 receptors on the blood vessels. Subsequently, antibodies accumulate at vascular walls with an ensuing immune reaction. Additionally, urticaria might be associated with bradykinin in the kinin-kallikrein system in conjunction with ACE2.^15^

There is no consensus on the definition of COVID-19 vesicular eruption. Reports from different countries manifest 4%‒15%^16^, ^17^ of adult patients experience COVID-19 vesicular eruption. In the pediatric patient population, this statistic was 4.3%. The pathogenesis of the vesicular exanthema remains to be unknown yet coincidence with herpes viruses has been observed.

In the present study, the authors have not encountered any chilblain-like lesions in patients with COVID-19. At the beginning of the pandemic, chilblain-like lesions on acral sites were suggested to be the main skin finding of the infection in outpatients who do not require hospitalization. Therefore, it has been assumed that chilblain-like lesions are indicators of mild COVID-19.^12^, ^18^ This conclusion might explain the absence of pernio-like lesions in the present study, considering the present study group was comprised of severe COVID-19 patients requiring hospitalization. Virus-induced type I interferonopathy, Thrombosis/coagulopathy, and Vasculitis caused by endothelial damage are proposed to be the main factors in the pathogenesis of the chilblain-like lesions. To elaborate, it has been postulated patients with chilblain-like lesions possess a stronger Interferon type one response to the SARS-CoV2, which attenuates the viral replication while causing vascular microangiopathic damage. The innate immune response, specifically the production of type one interferons, comprises the initial defense against viruses. The fact that patients with milder COVID-19 develop chilblain-lesions, proves their elevated Interferon type one levels prevent them from experiencing severe disease. COVID-19 patients have a predisposition for thromboembolism, which is demonstrated by elevated serum D-dimer levels and the presence of fibrin thrombi within capillaries in the lung and heart, occurrence of acral ischemic chilblain-like lesions. Microthrombi have also been documented in the chilblains-like lesions. Although vasculitis in COVID-19 can occur by hypoperfusion of the tissues, it has been proposed that SARS-CoV-2 can directly cause vasculitis. Angiotensin-Converting Enzyme (ACE)-2 has been suggested to be the membrane receptor of SARS-CoV-2, which might explain the positive immunostaining for SARS-CoV-2 in epithelial cells of eccrine glands.^15^

MIS-C is a disease precipitated by the SARS-CoV2 virus and characterized by persistent fever, the elevation of inflammatory markers, and single or multi-end-organ failure. Conjunctival and mucosal erythema, hand and foot edema, and coronary artery dilatation can be observed during the course of the disease, which is reminiscent of Kawasaki syndrome.^19^ The present study illustrated that patients with MIS-C are inclined to develop mucocutaneous findings (88.8%). In the MIS-C group, erythematous/maculopapular/morbilliform rash appears to be the most common pattern.

To summarize the relation between pediatric COVID-19 and skin manifestations; pediatric COVID-19 patients are more susceptible to developing mucocutaneous manifestations compared to adults. During the pandemic, clinicians should not overlook that the presence of high persistent fever and periorbital erythema and edema are predictive of MIS-C. When a child has a high persistent fever, respiratory symptoms, and a rash, COVID-19 should be excluded, and MIS-C should be kept in mind.

## Conclusions

On account of the high percentage of mucocutaneous findings, the authors propose COVID-19 should be acknowledged as one of the viral exanthem rashes of childhood because of due to the frequent occurrence of mucocutaneous findings.

## Financial support

None declared.

## Authors' contributions

Tunç Özen: Examined the skin, hair, nail, and mucosa of the inpatients; documented and photographed the cases; writing of the paper.

Filiz Cebeci Kahraman: Examined the skin, hair, nail, and mucosa of the inpatients; documented and photographed the cases; writing of the paper.

Sevliya Öcal: Diagnosis and treatment of COVID-19 inpatients.

Hüsnü Fahri Ovalı: Diagnosis and treatment of COVID-19 inpatients.

## Conflicts of interest

None declared.
